# Using Mobile Ecological Momentary Assessment to Understand Consumption and Context Around Online Food Delivery Use: Pilot Feasibility and Acceptability Study

**DOI:** 10.2196/49135

**Published:** 2023-11-29

**Authors:** Si Si Jia, Margaret Allman-Farinelli, Rajshri Roy, Philayrath Phongsavan, Karice Hyun, Alice Anne Gibson, Stephanie Ruth Partridge

**Affiliations:** 1 School of Health Sciences Faculty of Medicine and Health The University of Sydney Sydney Australia; 2 Nutrition and Dietetics Sydney School of Nursing The University of Sydney Sydney Australia; 3 Charles Perkins Centre The University of Sydney Sydney Australia; 4 Discipline of Nutrition and Dietetics Faculty of Medical and Health Sciences The University of Auckland Auckland New Zealand; 5 Prevention Research Collaboration Sydney School of Public Health The University of Sydney Sydney Australia; 6 Concord Hospital ANZAC Research Institute The University of Sydney Sydney Australia; 7 Menzies Centre for Health Policy and Economics The University of Sydney Sydney Australia

**Keywords:** ecological momentary assessment, mobile applications, mobile apps, feasibility studies, online food delivery, smartphone, young adult, adolescent, food environment, consumer behavior, mobile phone

## Abstract

**Background:**

Mobile ecological momentary assessment (EMA) is a powerful tool for collecting real-time and contextual data from individuals. As our reliance on online technologies to increase convenience accelerates, the way we access food is changing. Online food delivery (OFD) services may further encourage unhealthy food consumption habits, given the high availability of energy-dense, nutrient-poor foods. We used EMA to understand the real-time effects of OFD on individuals’ food choices and consumption behaviors.

**Objective:**

The primary aims of this pilot study were to assess the feasibility and acceptability of using EMA in young users of OFD and compare 2 different EMA sampling methods. The secondary aims were to gather data on OFD events and their context and examine any correlations between demographics, lifestyle chronic disease risk factors, and OFD use.

**Methods:**

This study used EMA methods via a mobile app (mEMASense, ilumivu Inc). Existing users of OFD services aged 16 to 35 years in Australia who had access to a smartphone were recruited. Participants were randomly assigned to 1 of 2 groups: signal-contingent or event-contingent. The signal-contingent group was monitored over 3 days between 7 AM and 10 PM. They received 5 prompts each day to complete EMA surveys via the smartphone app. In contrast, the event-contingent group was monitored over 7 days and was asked to self-report any instance of OFD.

**Results:**

A total of 102 participants were analyzed, with 53 participants in the signal-contingent group and 49 participants in the event-contingent group. Compliance rates, indicating the feasibility of signal-contingent and event-contingent protocols, were similar at 72.5% (574/792) and 73.2% (251/343), respectively. Feedback from the participants suggested that the EMA app was not easy to use, which affected their acceptability of the study. Participants in the event-contingent group were 3.53 (95% CI 1.52-8.17) times more likely to have had an OFD event captured during the study. Pizza (23/124, 18.5%) and fried chicken (18/124, 14.5%) comprised a bulk of the 124 OFD orders captured. Most orders were placed at home (98/124, 79%) for 1 person (68/124, 54.8%). Age (incidence rate ratio 0.95, 95% CI 0.91-0.99; *P*=.03) and dependents (incidence rate ratio 2.01, 95% CI 1.16-3.49; *P*=.01) were significantly associated with the number of OFD events in a week after adjusting for gender, socioeconomic status, diet quality score, and perceived stress levels.

**Conclusions:**

This pilot study showed that EMA using an event-contingent sampling approach may be a better method to capture OFD events and context than signal-contingent sampling. The compliance rates showed that both sampling methods were feasible and acceptable. Although the findings from this study have gathered some insight on the consumption and context of OFD in young people, further studies are required to develop targeted interventions.

## Introduction

### Background

Unhealthy diets are a leading contributor to the global burden of disease [1]. Globally, there is an increasing trend toward the consumption of “out-of-home” foods, potentially exacerbating suboptimal diets. These foods include fast foods and takeaway or “take-out” foods from a variety of sources including restaurants, fast-food chains, convenience stores, coffee shops, and takeaway food outlets. An analysis of the UK National Diet and Nutrition Survey 2008-2012 found that 27% of adults had consumed meals outside the home once per week or more [2], and research from the United States estimated that out-of-home meals comprised 50% of household budgets in 2018 [3]. Results from Australia’s National Household Expenditure and Time Use Surveys have shown a steady incline in spending on out-of-home foods—increasing from 22.8% of total food budgets in 1989 to 26.5% in 2010 [4]. Rising consumption of out-of-home foods has been similarly observed in low- and middle-income countries including China [5,6] and Latin American countries [7].

However, these out-of-home meals are often high in fat, salt, and sugar and low in vitamins and minerals [8]. A systematic review of 15 prospective studies found that a high frequency of eating out and consuming out-of-home meals was associated with weight gain and a greater risk of becoming overweight or obese [9]. Moreover, studies have shown associations between frequent consumption of home-cooked meals and greater adherence to the Dietary Approaches to Stop Hypertension diet and Mediterranean diets as well as greater intake of fruits and vegetables [10]. Thus, the replacement of home-cooked meals with out-of-home meals may result in further detriments to dietary health.

In recent years, online food delivery (OFD) services such as Uber Eats, DoorDash, and JustEat have transformed the concept of out-of-home foods by allowing customers to order a variety of food and drink items straight from kitchen to doorstep [11]. With this added convenience, OFD potentially increases access to and consumption of out-of-home food. A study from the United Kingdom found that adults with access to the greatest number of food outlets available to them online had 71% greater odds of OFD use (odds ratio [OR] 1.71, 95% CI 1.09-2.68) compared with those with the lowest access [12].

A growing number of studies have shown that a high proportion of menu offerings on popular OFD platforms are poor in nutritional quality. In Thailand, a majority of the most popular menu items were considered unhealthy against the World Health Organization’s recommended daily intake values [13]. Of the 25 most popular menu items, 23 exceeded the recommended sodium intake for adults, and 80% of all sweet items offered were 1.5 times above the recommended daily intake for sugar [13]. Similarly, more than three-quarters of menu items were classified as discretionary “junk foods” in Australia [14] and New Zealand [15]. A cross-sectional study conducted in 3 international cities (Chicago, Melbourne, and Amsterdam) revealed “burgers,” “pizza,” and “Italian” were in the top 10 most advertised meals on the website or app of the OFD [16].

OFD services are now regularly used by millions of people with heightened use found among young people aged between 16 and 34 years [17,18], who are already experiencing escalating rates of weight gain [19]. Furthermore, in Australia, by the age of 16 years, approximately 80% of young people have a debit card [20] in their name. This is critical considering the ease of digital payment options and tools offered on OFD apps, which amplifies young people’s accessibility to takeaway foods. A study in China found that >47.8% of male university students and >30.7% of female university students have used OFD more than once per week [21]. Therefore, there is an urgent need to assess and understand the consumption and behaviors associated with the use of these OFD services.

Traditional dietary assessment methods such as 24-hour recalls, diet history, and food frequency questionnaires are reliant on participants retrospectively recalling their intake, whereas food records involve prospective recording, but both approaches are time-consuming and prone to inherent biases [22,23]. Recent studies have demonstrated that ecological momentary assessments (EMAs) can be a valid measure of dietary intake [24,25]. EMA is a data collection method that gathers real-time information from participants studying behaviors and contexts as they perform regular day-to-day activities in real-world settings [26]. Due to technological advances, researchers now use smartphones or mobile phones to conduct EMA studies, which have improved data collection and added flexibility to study designs [27]. A systematic review of 39 EMA studies conducted in young people aged 16 to 30 years indicated that EMA is an acceptable and feasible methodology to capture dietary intake and food consumption for this population [28].

### Objectives

Although dietary intake generally can be spontaneous and difficult to capture, OFD events are likely to be even more sporadic. Thus, compared with traditional dietary assessment methods, EMA may be an alternate method for capturing OFD events. In addition, unlike traditional dietary assessment methods, the context including social and psychological factors surrounding the real-time OFD event can be obtained using EMA. To the best of our knowledge, no previous study has used EMA to study OFD use. Therefore, this study aimed to determine the feasibility and acceptability of EMA in a sample of young people who are users of OFD. A secondary aim was to investigate associations between the frequency of OFD use and demographic variables such as age, gender, and socioeconomic level as well as other lifestyle chronic disease risk factors including diet quality score, physical activity levels, stress levels, and sleep quality.

## Methods

### Recruitment

From June to October 2022, participants were recruited via flyers, social media advertising, and word of mouth. Eligible participants were (1) aged between 16 and 35 years; (2) users of OFD, defined as at least once in the past 3 months; (3) living in Australia; and (4) having access to a smartphone. This was to ensure that participants were not first-time users of OFD, as this study aimed to gain a specific understanding of the behaviors and demographics of OFD users. The target sample comprised at least 140 participants accounting for a 30% (42/140) dropout rate. Using quota sampling, the target was 28 participants in the 16 to 18 years age group, 28 participants in the 30 to 35 years age group, and 42 participants each in the 19 to 24 and 25 to 29 years age groups. Previous pilot EMA studies used nonprobability sampling methods to recruit a similar number of participants [25,29].

### Ethical Considerations

This study was approved by The University of Sydney Human Research Ethics Committee (2022/006). Reporting followed an adapted Strengthening the Reporting of Observational Studies in Epidemiology (STROBE) Checklist for Reporting EMA Studies (CREMAS) guidelines [30] (Multimedia Appendix 1).

### Data Collection

After participants joined the study and provided consent, they were directed to a series of baseline surveys on REDCap (Research Electronic Data Capture; Vanderbilt University) to obtain data on participant demographics, usual OFD behaviors, physical activity levels, sleep quality, and perceived stress levels. The questions were adapted from the 7-item International Physical Activity Questionnaire—Short Form [31], Pittsburgh Sleep Quality Index [30], and Perceived Stress Scale [32].

Upon completing the baseline surveys on REDCap, participants were sent a unique mobile code to download and set up the EMA mobile app (mEMASense, ilumivu Inc) on their personal smartphone devices. This app is available for both the Android and Apple operating systems. Using a computer-generated code, participants were randomly allocated 1:1 to 1 of 2 groups, “Prompts” or “No Prompts,” which determined the sampling approach used—respectively representative of the signal-contingent or event-contingent approach. A signal-contingent design involves sending prompts to participants randomly over the course of a given hour, day, or week [33]. In contrast, an event-contingent design allows participants to complete a prompt whenever they experience the event of interest [33].

Participants in each group received a separate set of instructions via REDCap to inform them of their group allocation and what was required from them in either the Prompts or No Prompts group. An external link was also sent to participants to complete the Australian Eating Survey [34], a validated food frequency questionnaire developed by researchers from the University of Newcastle, to capture usual dietary intake.

**Figure 1:**
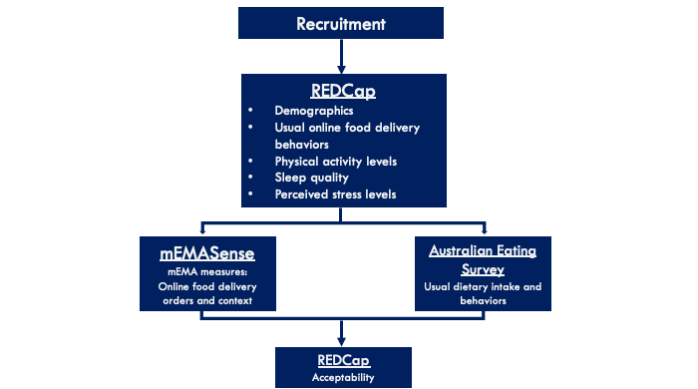
Flowchart of the data collection processes. mEMA: mobile-based ecological momentary assessment; REDCap: Research Electronic Data Capture.

**Figure 2:**
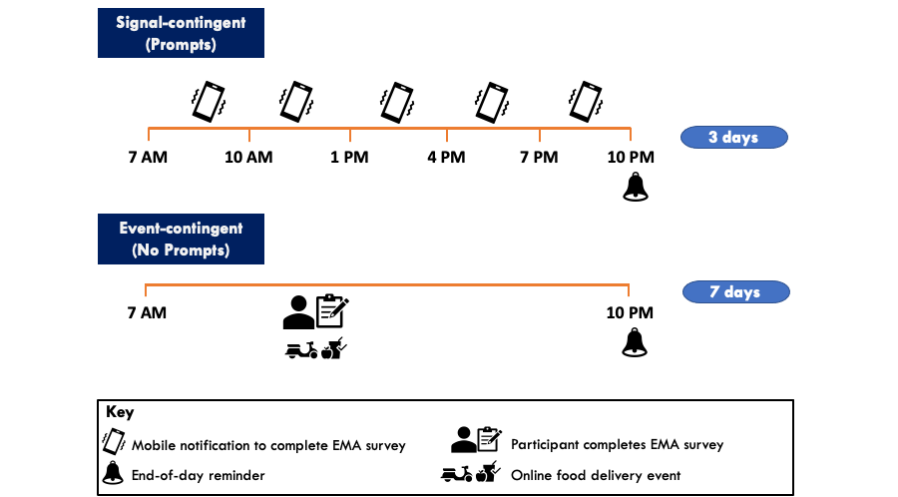
Overview of the 2 varying mobile-based ecological momentary assessment sampling protocols used in the study: signal-contingent (Prompts) group compared with the event-contingent (No Prompts) group. Participants in the No Prompts group were observed over 1 week (7 days), whereas those in the Prompts group were observed over 1 week with prompts sent on 3 separate days. EMA: ecological momentary assessment.

Data were collected over 1 monitoring period (1 wave). Figure 1 depicts a flow diagram of the overall data collection process, and Figure 2 provides an overview of the 2 varying EMA sampling protocols used in the study.

### EMA Procedure

#### Signal-Contingent Sampling (Prompts Group)

Participants in the Prompts group were monitored over 1 week on 3 days, including 2 weekdays (Monday-Friday) and 1 day on the weekend (Saturday or Sunday) between 7 AM and 10 PM. To ensure that at least 1 weekday and 1 weekend day were captured, the EMA prompt days were assigned on the second, fourth, and sixth day from the day participants joined the study. Participants were unaware of this prompting schedule and were only informed that they would be prompted on 3 “random days” over the 7 days from their enrollment into the study. This was implemented to improve the external validity of the study by aiming to capture unanticipated OFD events.

Participants were prompted by the app via a notification at a randomly assigned time during the 5 predetermined intervals (7 AM-10 AM, 10 AM-1 PM, 1 PM-4 PM, 4 PM-7 PM, and 7 PM-10 PM). An end-of-day prompt was sent at 10 PM on each of the study days to record any OFD ordering event that was not previously captured. When prompted, participants were asked to complete the short survey on the EMA app that started with the question “In the past 10-15 minutes, were you eating or drinking?” If unavailable at the time of the prompt, participants had a 45-minute time window to respond with 2 reminders sent 15 minutes apart after the initial prompt. After this point, the survey became inaccessible until the next prompt and recording opportunity. In total, 6 EMA surveys were prompted on each of the 3 study days, totaling 18 EMA surveys per participant across the data collection period.

#### Event-Contingent Sampling (No Prompts Group)

Participants in the No Prompts group were instructed to record any online food order they placed over 1 week from their enrollment in the study. An end-of-day prompt was sent at 10 PM on each of the 7 days to remind and allow participants to record any OFD event not previously captured.

#### Participants Attending High School

Participants aged between 16 and 18 years who were attending high school in the Prompts group were monitored across 3 days, including 2 weekdays (Monday-Friday) during nonschool hours (4 PM and 10 PM) and 1 day on the weekend (Saturday or Sunday) between 7 AM and 10 PM. This was done to ensure minimal disruption to high school commitments. Individuals attending high school in the No Prompts (the “event-contingent”) group were only required to answer the surveys during nonschool hours on weekdays (from 4 PM to 10 PM).

### Capturing an OFD Order Event

**Figure 3:**
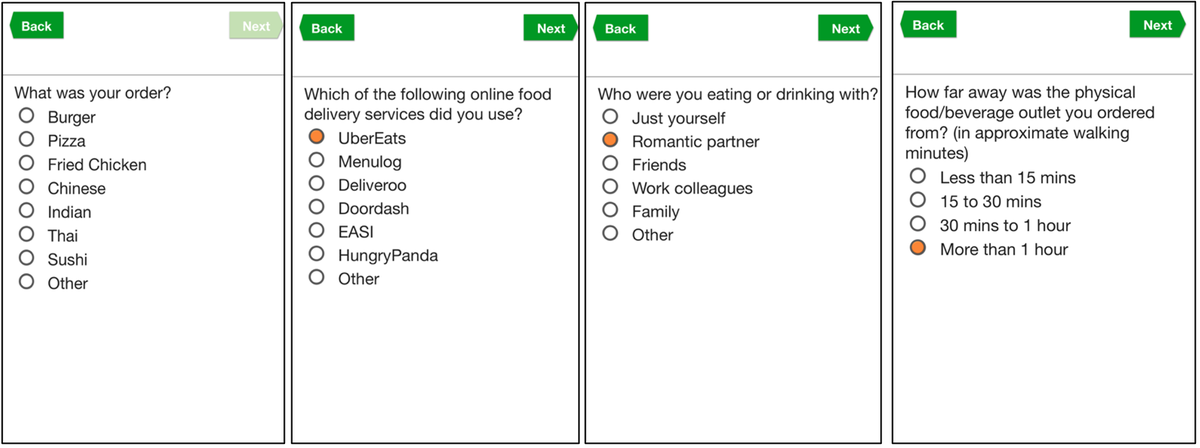
Selection of questions from the survey delivered through the mEMASense app to capture context surrounding an online food delivery event.

Participants in both the Prompts and No Prompts groups were asked for the following details and surrounding context of their online food order: how they received their order (whether through pick-up or delivery), what they ordered, who they were with (by themselves, with romantic partner, work colleagues, friends, or family), where they were (home, workplace, or university), how far away they were from the food outlet in walking minutes, if any promotions or discount codes were used, their hunger levels, and cravings and stress levels at the time of ordering. All instances were timestamped to record when they consumed their online food order. Please see Figure 3 for a screenshot of the EMA survey questions delivered through the mEMASense app.

### Incentives

Participants were offered gift vouchers up to AUD 45 (US $29) for their completion of the entire pilot study. If participants completed one-third of the study, they were awarded 1 AUD 15 (US $9) voucher for their time, and participants who had completed two-thirds were compensated with AUD 30 (US $19) worth of gift vouchers.

### Feasibility

The compliance rate was used to measure the feasibility of the study. This was calculated differently for the different sampling approaches. In the Prompts group, the compliance rate per participant was the number of answered prompts divided by 18, which was the total number of expected prompts. The mean compliance rate for the study was calculated to represent the sample in the Prompts group.

Similarly, for the No Prompts group, the compliance rate per participant was the number of answered end-of-day reminders divided by the total number of expected end-of-day reminders. These end-of-day reminders were sent daily over the 1-week study period, resulting in 7 total reminders. Furthermore, the percentage of online food ordering events captured by the end-of-day reminders was also analyzed. This indicated how likely participants were to forget their recording of each online food ordering event using the No Prompts approach.

### Acceptability

Participants were administered a final end-of-study questionnaire composed of 5 items to gather insight into their acceptability of the EMA methodology, the mobile app used to send the EMA prompts, and the perceived participant burden from the 2 different sampling approaches. These 5-point Likert scale questions were adapted from a previous study [29] with an additional closing open-ended question to gather general qualitative feedback.

### Study Measurements and Outcomes

#### Demographic

Age, gender, ethnicity, education status, working status, number of dependents, residential postcode, and car access were obtained from the demographic survey. Residential postcodes were matched to quintiles on the Index of Relative Socio-Economic Advantage and Disadvantage (IRSAD), an area-based socioeconomic measure, where a higher index value reflects an area of greater socioeconomic advantage compared with people residing in other areas [35]. Participants were also asked to report their height (cm) and weight (kg) so that their BMI could be determined.

#### Lifestyle Chronic Disease Risk Factors

Measures from the Australian Eating Survey generated the Australian Recommended Food Score, a diet quality index that reflects alignment with the Australian Dietary Guidelines [36]. Responses to the International Physical Activity Questionnaire allowed participants to be categorized into low, moderate, or high physical activity levels using scoring guidelines [37]. Similarly, a score was produced to categorize participants’ perceived stress levels into low, moderate, or high [38]. Sleep quality was reflected by the number of hours of sleep reported by the participants and compared with recommended sleep guidelines [39].

Data collected from the EMA app were used as the outcome variables of interest, including the number of OFD events, OFD order details, and related contextual data, as shown in Figure 1.

### Statistical Analysis

#### Overview

The study’s analytic sample comprised participants who met the minimum requirements outlined in the sampling protocol. This entailed completing at least one-third of the study period, which translated to a minimum of 4 prompts for adults or 2 prompts for adolescents within a single study day for those in the signal-contingent group. For participants in the event-contingent group, it meant answering at least 2 end-of-day reminders. In addition, those who had finished the end-of-study acceptability questionnaire were also included in this sample. Data sets from REDCap surveys, the Australian Eating Survey, and the EMA app were downloaded and linked together via participants’ unique mobile code for the EMA app and REDCap ID number. Data were checked and cleaned before analysis. All statistical analyses were performed using R statistical software (version 2023.03.0+386; R Core Team).

Descriptive statistics, including means, SDs, and frequencies, were used to report the baseline characteristics of the sample and the OFD behaviors and context captured via the EMA app. Other statistical analyses were conducted as described below in the Feasibility Analysis, Acceptability Analysis, Comparison of Sampling Approaches, and Exploratory Analysis sections.

#### Feasibility Analysis

Compliance rates were the main outcome to determine the feasibility of the EMA protocol. In the Prompts group (signal-contingent), this was calculated as the total number of prompts answered over the expected total number of prompts answered. These compliance rate calculations slightly differed for adolescent participants who received fewer prompts per study day. In the No Prompts group, as the event of interest may not have occurred daily, compliance was measured by participant’s responses to the end-of-day study reminders. This was similarly measured as the total number of end-of-day reminders answered above the expected total of 7.

To investigate factors associated with compliance, a 2-level multilevel logistic regression analysis was conducted on participant data from the Prompts group. The outcome variable used for analysis was whether the participant had answered the delivered prompt (coded as “TRUE”) or had not answered (coded as “FALSE”). Level 1 factors were factors at the observation level such as time of the day (ie, morning, afternoon, or evening) and day of the week (weekday or weekend). Level 2 factors were at the person or participant level such as their demographics including age, gender, and IRSAD decile. The intraclass correlation coefficients were calculated to determine the proportion of variance that is because of between-person differences.

#### Acceptability Analysis

Median scores and IQRs were reported to summarize responses to the 5-item acceptability questionnaire in both the Prompts and No Prompts groups. Open-ended feedback was analyzed using content analysis methods. Concepts were developed based on the occurrence of terms.

#### Comparison of Sampling Approaches

A multivariable logistic regression model was used to analyze whether the sampling approach (ie, Prompts vs No Prompts) was associated with capturing OFD events during the study period. The outcome variable was whether an OFD event was recorded during the study period. The model was adjusted for age, gender, and frequency of OFD ordering reported at baseline.

#### Exploratory Analysis on Demographic and Lifestyle Risk Factors and Their Associations With OFD Use

A zero-inflated negative binomial regression model was built to analyze associations between participant demographic characteristics, lifestyle chronic disease risk factors, and number of OFD events in a week. Incidence rate ratios were the effect size from this model used to indicate how often OFD events occurred over the study period in relation to participants’ demographic characteristics and lifestyle chronic disease risk factors. A zero-inflated model was deemed appropriate as our data had an excess of “zeros” for the number of OFD events recorded by the participants. This model was adjusted for age, gender, and socioeconomic status as indicated by the IRSAD quintile. An influential point, which is an outlier that affects the slope of the regression, was identified through a bar plot of the Cook distance and subsequently removed as it had a significant impact on the coefficients.

## Results

### Participants

**Figure 4:**
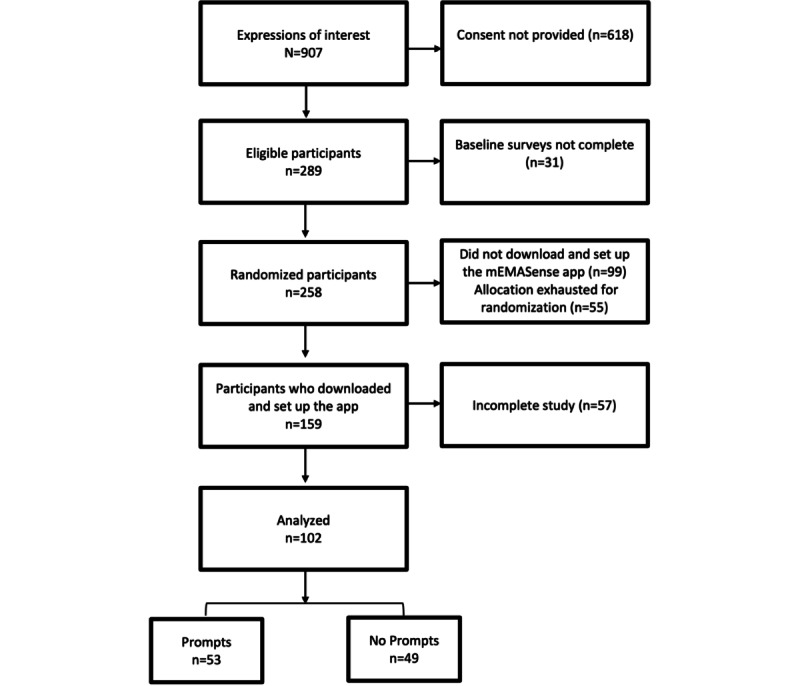
Participant recruitment process and attrition.

As shown in Figure 4, of the 907 expressions of interest received on REDCap, 289 participants provided consent and continued to complete the baseline surveys. However, only 258 (89.2%) of the 289 participants completed all baseline surveys and were subsequently randomized into either the Prompts or No Prompts group. Only 159 (61.6%) of the 258 participants proceeded to the next stage of the study by downloading and setting up the mEMASense app. Of the 159 participants, 102 (64.2%) were part of the final analytical sample, as 57 (35.8%) did not meet the minimum requirements for study completion. Of the 102 participants, there were 53 (52.0%) in the Prompts group and 49 (48.0%) in the No Prompts group.

### Participant Characteristics

The mean age of all participants was 24.4 (SD 5.4) years. Women comprised 70.6% (72/102) of the study sample, men comprised 28.4% (29/102) of the study sample, and 1.0% (1/102) of the participants identified as nonbinary. In total, 63.7% (65/102) of the participants had a normal BMI range, whereas 28.4% (29/102) were overweight and obese. Approximately half of the sample (48/102, 47%) identified their ethnicity as White, approximately a quarter (24/102, 23.5%) identified as Chinese, and 14.7% (15/102) reported their ethnicity as Asian and South-East Asian (not further specified). Most of the participants (89/102, 87.3%) resided in areas of high socioeconomic advantage in the upper quintiles of the IRSAD, and most participants (58/102, 56.9%) were highly educated (Table 1).

**Table 1:** Demographics of participants (N=102).

Demographics		Value
Age (years), mean (SD)		24.4 (5.4)
**Age (years), n (%)**		
	16-18	17 (16.7)
	19-24	34 (33.3)
	25-29	33 (32.3)
	30-35	18 (17.6)
**Gender identity, n (%)**		
	Woman	72 (70.6)
	Man	29 (28.4)
	Nonbinary	1 (1)
**BMI (kg/m^2^), n (%)**		
	Underweight (<18.5)	7 (6.9)
	Normal weight (18.5-24.9)	65 (63.7)
	Overweight (25.0-29.9)	20 (19.6)
	Obese (>30.0)	9 (8.8)
	N/A^a^	1 (1)
**Ethnicity, n (%)**		
	Asian (not specified)	11 (10.8)
	Bangladeshi	1 (1)
	Chinese	24 (23.5)
	Indian	3 (2.9)
	Japanese	1 (1)
	Jewish	1 (1)
	Southeast Asian (not further specified)	4 (3.9)
	Vietnamese	1 (1)
	White	48 (47)
	Multiracial^b^	7 (6.9)
	N/A^a^	1 (1)
**IRSAD^c^ quintiles (1 representing most disadvantaged and 5 representing most advantaged), n (%)**		
	Quintile 1	5 (4.9)
	Quintile 2	2 (2)
	Quintile 3	6 (5.9)
	Quintile 4	17 (16.7)
	Quintile 5	72 (70.6)
**Education level, n (%)**		
	Current high school student	13 (12.7)
	Completed high school	2 (2)
	Currently studying for degree or diploma	28 (27.4)
	Completed trade or technical qualification	0 (0)
	Completed a degree or diploma	33 (32.3)
	Completed a postgraduate degree	25 (24.5)
	N/A^a^	1 (1)
**Car access, n (%)**		
	Yes	61 (59.8)
	No	41 (40.2)
**Number of dependents, n (%)**		
	0	78 (76.5)
	1	8 (7.8)
	2	15 (14.7)
	3	1 (1)

^a^N/A: not applicable (owing to missing values).

^b^Including White and Chinese, White and Japanese, White and Pacific Islander, White and Asian, and nonspecified.

^c^IRSAD: Index of Relative Socio-Economic Advantage and Disadvantage.

### Usual OFD Behaviors and Lifestyle Behavioral Risk Factors

As shown in Table 2, in total, 52% (53/102) of the participants reported using an OFD service 1 to 2 times a month, and 27.5% (28/102) of the participants reported using it once a week at baseline. Approximately 89.2% (91/102) of the sample had used Uber Eats, which was followed by DoorDash as the next highly used service by 59.8% (61/102) of the participants. Only 23.5% (24/102) of the participants had membership with an OFD service, which was commonly a free 6-month trial.

The mean Australian Recommended Food Score for participants was 31 (SD 11.6) out of a maximum score of 73 indicating optimal nutrition. The median percentage of energy intake contributed by discretionary foods was 35% (IQR 26-44) among participants . Analyses from the International Physical Activity Questionnaire indicated that a large proportion of participants (64/102, 62.7%) had high physical activity levels, and 54.9% (56/102) of the sample experienced moderate levels of stress. The mean hours of sleep reported was 6.5 (SD 1.0) in adolescents and 7.2 (SD 1.1) in adults (Table 2).

**Table 2:** Online food delivery (OFD) behaviors at baseline and self-reported lifestyle behavioral risk factors (N=102).

Lifestyle risk factor				Value
**OFD behaviors**				
	**Frequency of using an OFD service, n (%)**			
		Rarely or never	0 (0)	
		1-2 times a month	53 (52)	
		Once a week	28 (27.4)	
		A few times a week	13 (12.7)	
		Once a day	4 (3.9)	
		More than once a day	4 (3.9)	
	**OFD service (those electing “Yes, I use this delivery service”), n (%)**			
		Uber Eats	91 (89.2)	
		MenuLog	58 (56.9)	
		Deliveroo	60 (58.8)	
		Doordash	61 (59.8)	
		EASI	18 (17.6)	
		Others^a^	12 (11.8)	
		Fantuan	3 (2.9)	
		HungryPanda	1 (1)	
		Panda	3 (2.9)	
		PandaFresh	2 (2)	
		Restaurant-specific delivery service	1 (1)	
		N/A^b^	3 (2.9)	
	**OFD services membership, n (%)**			
		Yes	24 (23.5)	
		No	78 (76.5)	
	**OFD membership (those electing “Yes, I have a membership subscription”)^a^, n (%)**			
		Dashpass	6 (5.9)	
		Deliveroo	3 (2.9)	
		Uber Eats	10 (9.8)	
		N/A^b^	5 (4.9)	
	**Monthly cost of OFD membership for those with a subscription (US $), n (%)**			
		Free trial for 6 months	6 (5.9)	
		Dashpass (9.99)	2 (2)	
		Uber Eats (9.99)	3 (2.9)	
		Deliveroo plus (12.99)	3 (2.9)	
		N/A^b^	10 (9.8)	
**Usual dietary behaviors^c^**				
	Australian Recommended Food Score (maximum 73), mean (SD)		31 (11.6)	
	Percentage of energy from noncore foods, median (IQR)		35% (26-44)	
	**Out-of-home food consumption (general, nonspecific to OFD), n (%)**			
		<1 per week	20 (19.6)	
		1-2 per week	51 (50)	
		3-4 per week	24 (23.5)	
		5-6 per week	3 (2.9)	
		Once a day	2 (2)	
		1 or more per day	2 (2)	
**Physical activity levels^d^ n, (%)**				
	Low		3 (2.9)	
	Moderate		35 (34.3)	
	High		64 (62.7)	
**Perceived stress levels^e^ n, (%)**				
	Low		30 (29.4)	
	Moderate		56 (54.9)	
	High		16 (15.7)	
**Hours of sleep, mean (SD)**				
	Adolescents aged 16-18 years (n=17)		6.5 (1.0)	
	Adults aged 18-35 years (n=85)		7.2 (1.1)	

^a^Participants were allowed to enter >1 other food delivery service or membership they used.

^b^N/A: not applicable (owing to invalid online food delivery service).

^c^Obtained from the Australian Eating Survey results.

^d^Physical activity levels were calculated according to the International Physical Activity Questionnaire analysis guidelines [37].

^e^Perceived stress levels were calculated according to guidelines for the Perceived Stress Scale [38].

### Feasibility

Compliance rate was used as a measure of feasibility for the EMA study. Among the analytic sample, compliance with the study protocol was compared between the Prompts and No Prompts groups.

#### Prompts Group

##### Adolescents Aged 16-18 Years

The compliance rate for the adolescent Prompts group was 70.4% (76/108), with an average of 8 (out of 12) prompts answered per adolescent participant.

##### Adults Aged 18-35 Years

The compliance rate for the adult Prompts group was 72.5% (574/792), with an average of 13 (out of 18) prompts answered per adult participant.

#### No Prompts Group

Compliance in the No Prompts group was measured by the response to all end-of-day study reminders. The compliance rate for the No Prompts group was 73.2% (251/343). Furthermore, of 49 participants, 36 (73%) followed the protocol and logged instances of using an OFD service during their study period, unprompted, as per the “event-contingent” sampling protocol.

### Factors Influencing Missing Prompt Data

Results from a multilevel logistic regression model showed that compliance or response to a prompt did not vary by age, gender, socioeconomic status, or whether it was a weekday or weekend (Table 3). However, compared with the evening between 6 PM and 12 AM, participants were 1.47 times more likely to answer prompts in the afternoon between 12 PM and 6 PM (95% CI 1.02-2.12). The intraclass correlation coefficients between participants were low (0.27), indicating that multilevel modeling was appropriate.

**Table 3:** Results from the multilevel logistic regression model to examine observation level and within-person level factors and their influence on a participant’s likelihood to respond to prompts (n=53).

Predictors	Response to prompt (yes or no)	
	Odds ratios (95% CI)	*P* value
Days (weekend)	0.81 (0.59-1.11)	.18
Time of day (afternoon)	1.47 (1.02-2.12)	.04^a^
Time of day (morning)	0.84 (0.58-1.22)	.36
Age (years)	1.02 (0.95-1.08)	.6
Gender (woman)	2.07 (1.00-4.31)	.05
IRSAD^b^ decile	1.19 (0.99-1.42)	.06

^a^Intraclass correlation coefficient: 0.27.

^b^IRSAD: Index of Relative Socio-economic Advantage and Disadvantage.

### Acceptability

#### Prompts

Responses from participants in the Prompts group reflected an overall opinion that the number of prompts sent during the study period was an appropriate amount, indicated by a median score of 3. The times of day that the prompts were sent were generally agreed upon by participants as being appropriate and not burdensome, with a high median score of 4 (Multimedia Appendix 2). Participants were ambivalent in their response to the app being easy to use, with a median score of 3 indicating that they neither agreed nor disagreed with the statement. The EMA study did not appear to make a majority of participants more conscious of their diet and eating behaviors, with more responses skewed toward disagreeing with the statement. The length of study for the Prompts group, however, was distributed more toward being *too short* (Multimedia Appendix 2).

#### No Prompts

In the No Prompts group, participants’ responses also showed that the number of prompts sent during the study period was appropriate, with a median score of 3. Many participants agreed that end-of-day reminders were necessary to remember to log an OFD order, as shown by the median score of 4 and with a distribution skewed toward “strongly agree” (Multimedia Appendix 2). However, more participants disagreed that the mobile app was easy to use. This was indicated by a median score of 3, with more scores indicating strong disagreement. Furthermore, responses from participants varied on whether the surveys made them more conscious of their diet and eating behaviors, as shown by the median score of 3. Responses on length of study for the No Prompts group were also distributed more toward being *too short*.

#### Open Feedback

Open feedback was obtained from 45 participants. Responses from 38% (17/45) of the participants indicated the difficulty of navigating the app, with many stating that it was “clunky,” “not user-friendly,” and “cumbersome.” Moreover, 27% (12/45) of the responses indicated that the timing of the prompts could be improved. Participants in the group that received prompts suggested receiving prompts closer to mealtimes. Some participants in the No Prompts group commented on the timing of the “end of day” reminders as being “too late” and after they had gone to sleep.

Moreover, 4 participants suggested further clarity of the training instructions and to have these instructions available and accessible to them throughout the study period. There were variable open responses on study length, although more participants wrote that a longer study period would be more representative and able to capture their OFD behaviors.

### OFD Events

#### Accuracy of Using EMA to Capture OFD Events

As shown in Table 4, this study captured 124 OFD events from 56.9% (58/102) unique participants. The actual frequency of OFD ordering throughout the study period did not match the reported frequency at baseline in 52% (53/102) of the participants and exactly matched in 48% (49/102) of the participants. For instance, if a participant had reported using an OFD service once a week in the baseline survey and the study did not capture an OFD event during the monitoring period, this would be a case where the reported frequency did not match the actual frequency. In the 53 participants where actual food ordering frequency differed, 26 (49%) had more OFD events during the study compared with 27 (51%) who had ordered less than what was reported at the start of the study.

**Table 4:** Comparison of the number of online food delivery (OFD) events captured between varying sampling approaches (event-contingent vs signal-contingent; n=58 unique participants).

Sampling approach	OFD events captured, n	OFD orders from end-of-day reminders, n	Total, n
No Prompts (event-contingent)	57	22	79
Prompts (signal-contingent)	41	4	45
Total	98	26	124

As shown in Table 4, there appeared to be more OFD events (79/124, 63.7%) captured from the event-contingent group compared with the signal-contingent group (45/124, 36.2%). Results from a multivariable logistic regression showed that there was a strong level of evidence to support a significant association between the sampling approach and the capture of an OFD event during the study period after adjusting for age and gender. Compared with the Prompts group, participants in the No Prompts group were 3.53 times more likely (95% CI 1.52-8.17) to have logged an OFD event.

#### Contextual Data Surrounding Online Food Ordering Events

A total of 124 instances of OFD were captured from 58 unique participants. More than half of all orders (65/124, 52.4%) were placed in the evening (between 6 PM and 12 AM) and were less common at night or morning, comprising 9.7% (12/124) of ordering events each (Multimedia Appendix 3). As shown in Multimedia Appendix 3, most orders were made on a Saturday (25/124, 20.2%), followed by Tuesday (22/124, 17.7%) and Friday (21/124, 16.9%). The most common food delivery orders were pizza (23/124, 18.5%), fried chicken (18/124, 14.5%), Chinese food (18/124, 14.5%), and burgers (17/124, 13.7%). However, 28.2% (35/124) of the orders were from a category other than what was provided, including Mexican; bubble tea; fast-food franchises (Red Rooster, KFC, and McDonald’s); cakes, pastries, and bakery items; groceries; and Vietnamese food. The following food categories were mentioned only once from all OFD events: hot chips and Caesar salad, breakfast platter, Malaysian, Nepalese, Afghan, modern Australian, Japanese, fried vegetables, Korean (fried chicken, chips, and seafood pancake), salad bowl, and sandwich.

For 50% (62/124) of the OFD orders captured, participants used Uber Eats, followed by DoorDash (23/124, 18.5% of the orders). In 54.8% (68/124) of the orders, participants ordered for just themselves, 15.3% (19/124) ordered for family, and 13.7% (17/124) ordered for their romantic partner.

The prevailing reason for using an OFD service was convenience, which contributed to 37.1% (46/124) of the responses. Taste and cravings attributed to 20.9% (26/124) of the orders. Most participants (98/124, 79%) ordered when they were at home, and 8.1% (10/124) of the participants ordered when they were at university or technical and further education. In 54.8% (68/124) of the online food ordering events, participants had ordered from a food outlet situated within a 15- to 30-minute walking distance, and 21.8% (27/124) of the participants ordered from an outlet that was between a 30-minute to 1-hour walk away (Multimedia Appendix 3).

A promotional code or offer was used for approximately one-third (37/124, 29.8%) of all orders. A wide variety of promotions and discounts were reported by the participants, from free delivery to receiving 10% or 50% off on their orders. The following discounts and promotions were only mentioned once from all OFD events: first-order discount, free additional food, referral discount, ShopBack, 30% off, AUD $16 off, AUD $20 off, and AUD $30 off (AUD $1 = USD $0.66). For 50.8% (63/124) of recorded OFD events, participants described the hunger levels as “quite hungry” or “really hungry.” In 72.6% (90/124) of the OFD orders, participants responded that the order satisfied their cravings. Participants reported neutral to high levels of stress, ranging from not stressed or relaxed to very stressed in 72.6% (90/124) of all the OFD events.

### Associations

In a zero-inflated negative binomial regression model adjusted for age, gender, and socioeconomic status, there was evidence that age and dependents were significantly associated with the number of OFD events in a week (Table 5). With every 1-year increase in age, the incidence rate of OFD events in a week decreased by 5% (95% CI 0.91-0.99). Compared with participants with no dependents, those with dependents had an incidence rate of OFD events 2.01 times higher (95% CI 1.16-3.49). Stress levels, age, gender, and socioeconomic status were not statistically significant factors associated with OFD events.

**Table 5:** Incidence rate ratios (IRRs) of demographic and lifestyle risk factors in a zero-inflated negative binomial regression model (N=101).

Factors		IRR (95% CI)	*P* value
**Dependents**			
	No dependents (reference)	N/A^a^	N/A
	Dependents	2.01 (1.16-3.49)	*.01*
	Diet quality score	0.98 (0.96-1.00)	.1
**Stress levels**			
	High stress (reference)	N/A	N/A
	Moderate stress	0.60 (0.36-1.00)	.05
	Low stress	0.51 (0.26-1.00)	.05
Age (years)		0.95 (0.91-0.99)	.03
**Gender**			
	Man (reference)	N/A	N/A
	Woman	0.85 (0.47-1.54)	.59
Socioeconomic status (IRSAD^b^)		0.97 (0.84-1.13)	.73

^a^N/A: not applicable.

^b^IRSAD: Index of Relative Socio-Economic Advantage and Disadvantage.

## Discussion

### Principal Findings

This pilot study tested the feasibility and acceptability of using EMA to capture OFD events and contextual behaviors in young people aged 16 to 35 years. Compliance rates, as an indicator of feasibility, to signal-contingent and event-contingent protocols were similar at 72% and 73%, respectively, in adults. However, participants in the event-contingent group were more likely to have captured an OFD event during the study period. The EMA protocols for both groups were acceptable; however, most participants agreed that the study could be improved by an app that was easier to navigate. Contextual data revealed that OFD orders consisted largely of unhealthy foods. These orders were commonly placed for evening meals, typically after 6 PM, were mostly for a single person, and were ordered at home. Moreover, the orders often originated from an outlet within a 15- to 30-minute walking distance. Analyses showed that there was a significant association between age, number of dependents, and number of OFD events. These formative findings warrant further investigation with refinement of the EMA methodology to increase acceptability and improve the OFD data captured from participants.

High compliance rates indicated a feasible and acceptable EMA protocol for the study population and events captured. In the guidelines proposed by Stone and Shiffman [40] for EMA protocols, compliance rates of ≥80% are considered optimal. This study observed compliance rates between 70% and 73%, which fell short of the recommendations yet aligned with the findings from systematic reviews of similar dietary EMA studies conducted in young people. In a review by Liao et al [30], an average compliance rate of 71% was reported among studies. However, this is in contrast with the review by Battaglia et al [28], where compliance rates were reported to be >80% in more than half of the included studies. Stone et al [41] suggested that noncompliance is mainly attributed to monitoring burden and participants forgetting to record. For this study, the 3 additional reminders set 15 minutes apart and 1 end-of-day prompt included in the protocol aimed to eliminate instances of noncompliance from forgetfulness. Therefore, the monitoring burden may have influenced compliance. Feedback from participants showed that 27% (12/45) of the participants thought that the timing of prompts could have been improved. Data from the multilevel regression analysis supported this as prompts were more likely responded to in the afternoon compared with the evening. Similarly, another feasibility study on a mobile EMA intervention reported that several participants disliked receiving alerts at night, which may have influenced response rates to prompts [42]. These are important considerations for future EMA study designs aiming to collect data from participants during evening hours.

Participants who were assigned to the event-contingent sampling approach were 3 times more likely to have had an OFD event captured during the study compared with the signal-contingent group. This indicates that event-contingent sampling appeared to be the better approach to capturing OFD events. It is likely that event-contingent sampling was more appropriate because of OFD being a specific and sporadic event that, among study participants, varied in the frequency of use. Thus, unprompted records of any instance of OFD over the 1-week monitoring period were more effective than answering prompts delivered on 3 random sampling days. However, an additional end-of-day reminder proved to be critical for the event-contingent group as almost one-third (22/79, 28%) of the OFD orders were captured through this reminder, which otherwise would have been forgotten.

Another possible key factor affecting participants’ experience of the EMA study and compliance was the perceived ease of use of the mobile app used to capture data. Many participants found that the EMA app was difficult to navigate and not user friendly, which may have negatively affected their experience of this study. Another EMA study also found participants commenting on the “difficult and bothersome” nature of EMA apps, although this was specific to the Visual Analog Slider used for one of the momentary assessments [29]. The Technology Acceptance Model is a highly influential information systems theory that suggests consumer acceptability is largely attributed to the perceived ease of use and usefulness of a given technological tool [43]. Consequently, the layout and technical aspects of EMA app themselves are critical to improving acceptability and compliance by participants.

This study revealed that orders were commonly placed for evening meals, typically after 6 PM, were mostly for a single person, and were ordered at home. Moreover, the orders often originated from an outlet within a 15- to 30-minute walking distance. According to physical activity research, a 1-km distance typically reflects a 15- to 20-minute walk for an average adult [44]. As such, this study revealed that a large proportion of customers are ordering food from potentially >1-2 km away and beyond what is typically considered their walkable neighborhood food environment [45] or the 20-minute neighborhood concept that is growing in popularity [46]. Furthermore, the results from this study are consistent with an annual market report from Uber Eats Canada, which revealed that 6 PM, typically considered “dinner time,” was the most popular time of day for orders [47]. An Australian study on the food preparation location context of meals and snacks consumed by young adults showed that dinners prepared from outside of the home are predominately discretionary (20%) compared with the predominately 5-food group (13%) [48]. In addition, pizza was the most popular food category that was ordered, followed by fried chicken, Chinese food, and burgers. These findings also align with the annual report from Uber Eats Australia [49], which indicated that chicken burgers and chicken burritos were in the top 10 most popular orders list from local small businesses. Altogether, this provides preliminary evidence that most foods ordered by OFD services are not healthy and supplements previous studies that have analyzed online menu items and showed that a high proportion of offerings are poor in nutritional quality [13-15,50].

The results from a zero-inflated negative binomial regression model showed that dependents were positively associated with the number of OFD events. This finding is consistent with a previous study conducted by Keeble et al [51], which determined that the odds of any OFD service use were greater for those living with children (OR 2.71, 95% CI 2.44-3.01). Similarly, another study established that the positive relationship between access to the greatest number of food outlets online and use of OFD services in the previous week was specific to those living with children [12]. It is possible that dependents may increase their levels of financial and time stress [52], both of which present barriers to the preparation of healthy home-cooked meals [53]. The use of OFD services may help alleviate time constraints and hence explain the higher frequency of use among those with dependents.

Convenience prevailed as the selected reason for an OFD order (46/124, 37.1%), whereas “being busy” accounted for 12.1% (15/124) of the orders. Price promotions were also used in approximately one-third of all recorded events. These findings align with a qualitative study from the United Kingdom that investigated customer experiences of using OFD services [54]. Keeble et al [54] identified “less effort for more convenience” as a key theme behind customer’s use of OFD. In addition, price promotions often influenced and justified the use of these services. Individuals with limited time may be higher consumers of out-of-home food, as research from the United States showed that in a sample of busy young adults, working >40 hours a week was associated with time-related barriers to healthful eating [55]. This perceived lack of time is linked to lower fruit and vegetable intake and greater intake of convenience and fast foods [56]. Of concern, a previous study has shown that ordering meals online for home delivery was significantly associated with higher levels of sugary drinks consumption (*P*=.003) and fast-food restaurant patronage (*P*<.001) [57]. A survey conducted in China similarly showed that young adults aged 18 to 30 years often ignored the nutritional value of their OFD orders; however, concerning trends emerged, as these young consumers reported potential physical health changes such as weight gain, elevated blood lipids, and gastrointestinal discomfort as a result of long-term OFD use [58]. However, this study did not find a significant association between diet quality score and the number of OFD events. Despite this, further research is needed to clarify the relationship between unhealthy diets and OFD use.

### Strengths and Limitations

To the best of our knowledge, this is the first study to use EMA to capture OFD behaviors and context in near real time. Real-time monitoring is critical to ensuring ecological validity and is a major advantage of the EMA methodology, given the sporadic nature of OFD use. Moreover, this study gathered data on the use of OFD that is independent of market research or company reports and is an important addition to the public health literature that currently lacks evidence on the consumption of OFD.

However, several limitations of this study must also be acknowledged. This was a pilot study where 2 varying EMA protocols were tested for their feasibility and acceptability. Hence, caution is warranted when interpreting the associations found in this study between demographics, lifestyle risk factors, and OFD events. In addition, this study has yet to validate the capture of OFD consumption against gold standard dietary assessment methods such as the 24-hour recall. An important next step would be to examine the percentage match for each occasion of OFD consumption between a 24-hour recall and the EMA protocol.

All data gathered and analyzed in this study were self-reported by the participants, which is inherently prone to response bias. Furthermore, the EMA app used in this study was limited to iOS or Android users and appeared to cause more issues with Android users. Owing to convenience sampling, the study sample was highly educated females residing in areas of high socioeconomic advantage, which accordingly may limit the generalizability of the findings.

In addition, it is noted that the monitoring period of 7 days may not have adequately or accurately captured OFD events, as more than half of the study sample reportedly used OFD services 1 to 2 times a month. Future studies could use the event-contingent sampling approach and potentially extend the monitoring period to 14 days instead, alongside incorporating end-of-day reminders.

Moreover, although a training guide was provided online to participants, the study would also benefit from an in-person training day, as recommended by previous EMA studies [59,60]. This would reinforce what is required of the participants for the study, reduce missing data, and improve adherence to the protocol. A training day can also provide an opportunity to obtain objective measurements for weight, height, and waist circumference.

### Conclusions

This study showed that mobile EMA is a viable method to capture OFD events and behaviors of its users in near real time, with better results obtained from event-contingent sampling. However, further amendments to the study protocol are necessary to improve compliance and acceptability among participants. In addition, validation of the EMA protocol against gold standard dietary assessment methods such as the 24-hour recall is an important next step. Further research is also essential to support the significant associations found between higher OFD use and those of younger age and those with dependents. This formative work may inform future EMA protocols to capture OFD orders and contextual factors and assist in further understanding OFD use and its impact on diet and health.

## Appendix

Multimedia Appendix 1 Adapted Strengthening the Reporting of Observational Studies in Epidemiology (STROBE) Checklist for Reporting EMA Studies (CREMAS).

Multimedia Appendix 2 Acceptability of the mobile-based ecological momentary assessment protocol comparing the 2 sampling approaches.

Multimedia Appendix 3 Table of online food delivery (OFD) behaviors and contextual data captured from 58 unique participants.

## Abbreviations

EMA: ecological momentary assessment
IRR: incidence rate ratio
IRSAD: Index of Relative Socio-Economic Advantage and Disadvantage
OFD: online food delivery
REDCap: Research Electronic Data Capture
