# Supplementary material for: Using Mobile Ecological Momentary Assessment to Understand Consumption and Context Around Online Food Delivery Use: Pilot Feasibility and Acceptability Study
Source: JMIR Mhealth Uhealth. 2023 Nov 29;11:e49135. doi: 10.2196/49135 (PMC10719819; doi:10.2196/49135)
Supplement: Multimedia Appendix 1 [file mhealth_v11i1e49135_app1.pdf]

Supplementary Table 1. Checklist for Reporting Ecological Momentary Assessment Studies[a]

	Item Number	Recommendation	Line
Title	1	Include ecological momentary assessment in title and key words	Title, Keywords
**Introduction**			
Rationale	2	Briefly introduce the concept of EMA and provide reasons for utilizing EMA for this study or topic of interests (eg, to examine time-varying predictors of unhealthy eating occasions in children's daily lives)	Introduction, Line 109-110, 117-122
**Methods**			
Training	3	Indicate if, and by what methods, training of participants for EMA protocol was used	Methods, Line 166-168
Technology	4	Describe what technology, if any, was used. Include the following information: device (eg, mobile phone, portable computer), model (eg, Nexus 4, iPod), operating system (eg, Android, Windows), and EMA program name	Methods, Line 156-157
Wave duration	5	State the number of waves for the study (eg, 2 monitoring periods over the course of 1 year)	Methods, Line 168
Monitoring period	6	State the number of days each wave of the study lasted, and how many weekdays versus weekend days	Methods, Line 171-209
Prompting design	7	Indicate the prompting strategy used for the study (eg, event-based, interval-based, or a combination of the two). If using interval-based strategy, indicate what type of schedule is used (eg, fixed, random, or hybrid interval)	Methods, Line 171-209
Prompt frequency	8	Intended frequency of prompts per day. Break down by weekdays and weekend days if applicable	Methods, Line 171-209
Design features	9	Describe any design feature to address potential sources of bias (eg, reactivity) or participant burden (eg, EMA questions appearing in different orders)	Methods, Line 189-192
**Results**			
Attrition	10	Indicate participant attrition throughout the study; report attrition rates both by monitoring days and waves, if applicable	Results, Figure 4, Line 327-334
Prompt delivery	11	Report number of EMA prompts that were planned to be delivered. If possible, also report the number of EMA prompts	Results, Line 387-394

		that were actually received by participants and indicate reasons for why prompts were not sent out (eg, technical issues or participant noncompliance reason such as phone was powered off)	
Latency	12	Report the amount of time from prompt signal to answering of prompt	N/A
Compliance rate	13	Report total answered EMA prompts across all subjects and the average number of EMA prompts answered per person. Report compliance rate both by monitoring days and waves, if applicable. Indicate reasons for noncompliance, if known	Results, Line 387-401
Missing data	14	Report whether EMA compliance is related to demographic or time-varying variables	Results, Line 402 - 407, Table 3
**Discussion**			
Limitations	15	Discuss limitations of the study, taking into account sources of potential bias when using EMA methods (eg, reactivity, use of technology)	Discussion, Line 678-703
Conclusions	16	Provide a general interpretation of results and discuss the benefits of using EMA (eg, improving understanding of daily behaviors)	Conclusion, Line 706-708

[a]Table adapted from Kracht, C.L., Beyl, R.A., Maher, J.P. *et al.* Adolescents' sedentary time, affect, and contextual factors: An ecological momentary assessment study. *Int J Behav Nutr Phys Act* **18**, 53 (2021). https://doi.org/10.1186/s12966-021-01121-y **based on** Liao Y, Skelton K, Dunton G, Bruening M. A systematic review of methods and procedures used in ecological momentary assessments of diet and physical activity research in youth: An adapted STROBE checklist for reporting EMA studies (CREMAS). J Med Internet Res. 2016;18(6):e151. https://doi.org/10.2196/jmir.4954.
