# Supplementary figures and images for: Using Mobile Ecological Momentary Assessment to Understand Consumption and Context Around Online Food Delivery Use: Pilot Feasibility and Acceptability Study
Source: JMIR Mhealth Uhealth. 2023 Nov 29;11:e49135. doi: 10.2196/49135 (PMC10719819; doi:10.2196/49135)

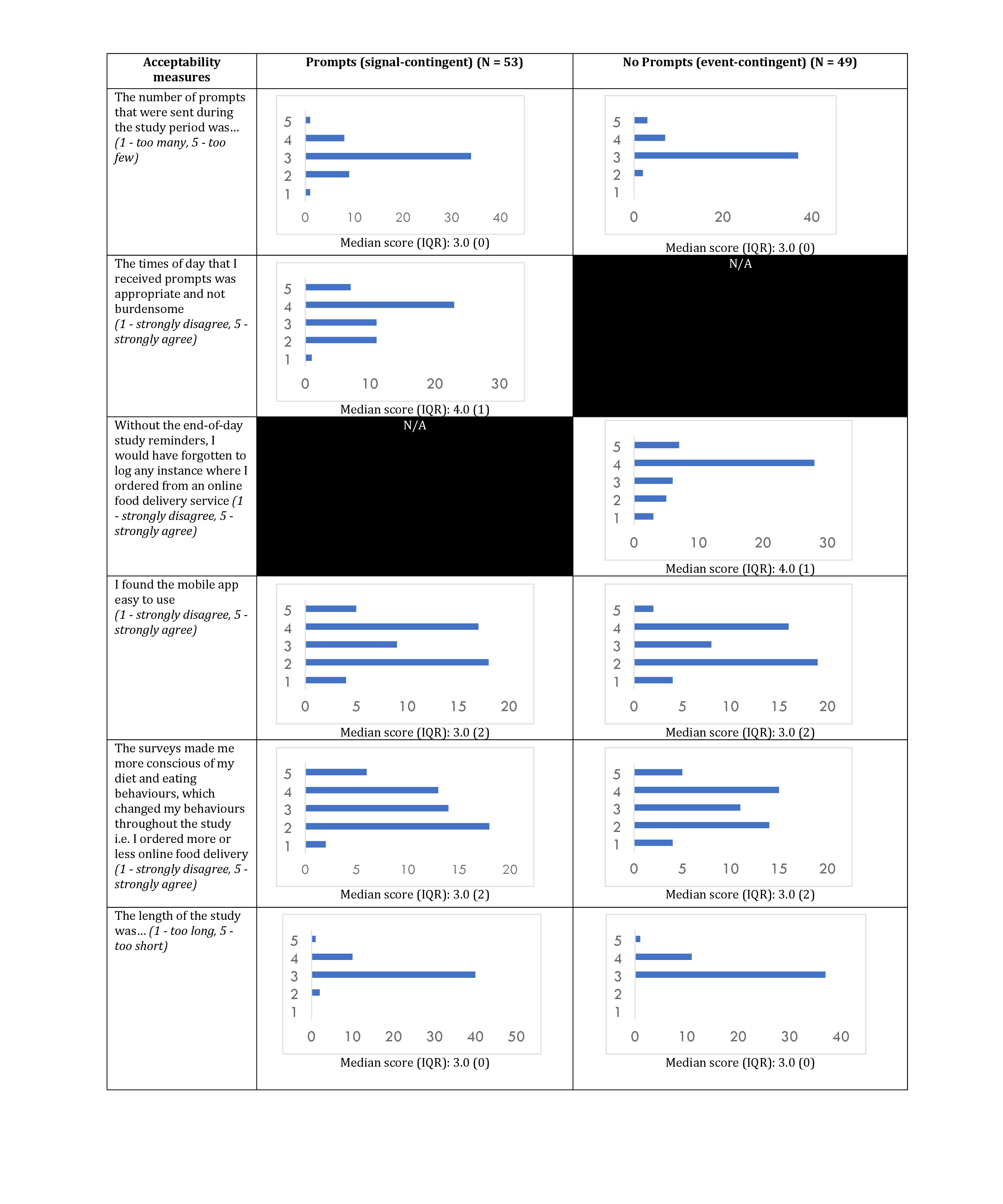

Supplement: Multimedia Appendix 2 [file mhealth_v11i1e49135_app2.png]
