# Supplementary material for: Using Mobile Ecological Momentary Assessment to Understand Consumption and Context Around Online Food Delivery Use: Pilot Feasibility and Acceptability Study
Source: JMIR Mhealth Uhealth. 2023 Nov 29;11:e49135. doi: 10.2196/49135 (PMC10719819; doi:10.2196/49135)
Supplement: Multimedia Appendix 3 [file mhealth_v11i1e49135_app3.docx]

**Multimedia Appendix 3.** Table of online food delivery (OFD) behaviors and contextual data captured from 58 unique participants.

| **Characteristics of OFD orders** | | Value |
| --- | --- | --- |
| Online food ordering events captured, N | | 124 |
| Number of OFD orders during study period per participant, mean (SD) | | 2.1 (1.9) |
| **Time of day, n (%)** | | |
|  | Morning (6 AM to 12 PM) | 12 (9.7) |
|  | Afternoon (12 PM to 6 PM) | 35 (28.2) |
|  | Evening (6 PM to 12 AM) | 65 (52.4) |
|  | Night (12 AM to 6 AM) | 12 (9.7) |
| **Weekday, n (%)** | | |
|  | Monday | 10 (8.1) |
|  | Tuesday | 22 (17.7) |
|  | Wednesday | 12 (9.7) |
|  | Thursday | 20 (16.1) |
|  | Friday | 21 (16.9) |
|  | Saturday | 25 (20.2) |
|  | Sunday | 14 (11.3) |
| **Food order category, n (%)** | | |
|  | Burger | 17 (13.7) |
|  | Pizza | 23 (18.5) |
|  | Fried chicken | 18 (14.5) |
|  | Chinese | 18 (14.5) |
|  | Sushi | 7 (5.6) |
|  | Indian | 3 (2.4) |
|  | Thai | 3 (2.4) |
|  | Other | 35 (28.2) |
|  | Mexican | 11 (8.9) |
|  | Bubble tea | 3 (2.4) |
|  | Fast-food franchise (Red Rooster, KFC, and McDonald’s) | 3 (2.4) |
|  | Cakes, pastries, and bakery items | 3 (2.4) |
|  | Groceries | 2 (1.6) |
|  | Vietnamese | 2 (1.6) |
|  | Other (varied)^a^ | 11 (8.9) |
| **OFD service, n (%)** | | |
|  | Uber Eats | 62 (50.0) |
|  | DoorDash | 23 (18.5) |
|  | HungryPanda | 9 (7.3) |
|  | Deliveroo | 7 (5.6) |
|  | Menulog | 5 (4) |
|  | EASI | 2 (1.6) |
|  | Other | 7 |
|  | WeChat takeaway | 2 (1.6) |
|  | PandaFresh | 2 (1.6) |
|  | HelloFresh | 1 (0.8) |
|  | Tuanyuan | 1 (0.8) |
|  | Fantuan | 1 (0.8) |
|  | Direct from café or restaurant | 9 (7.3) |
|  | Domino’s | 3 (2.4) |
|  | KFC | 1 (0.8) |
|  | Crust Pizza | 1 (0.8) |
|  | Pizza Hut | 1 (0.8) |
|  | Starbucks | 1 (0.8) |
|  | Unspecified café/restaurant name | 2 (1.6) |
| **Number of people the order catered for, n (%)** | | |
|  | Just yourself | 69 (55.6) |
|  | 2 | 29 (23.4) |
|  | 3 | 9 (7.3) |
|  | ≥4 | 12 (9.7) |
|  | ^__ b^ | 5 (4.0) |
| **Whom the order catered for, n (%)** | | |
|  | Just yourself | 68 (54.8) |
|  | Romantic partner | 17 (13.7) |
|  | Friends | 14 (11.3) |
|  | Work colleagues | 1 (0.8) |
|  | Family | 19 (15.3) |
|  | Other | 0 (0) |
|  | ^__ b^ | 5 (4.0) |
| **Reason for ordering OFD, n (%)** | | |
|  | Convenient | 46 (37.1) |
|  | Taste/cravings | 26 (20.9) |
|  | Busy | 15 (12.1) |
|  | Unmotivated to cook | 12 (9.7) |
|  | Price (cheap, transparent pricing, and discount) | 10 (8.1) |
|  | Social celebration/special treat | 7 (5.6) |
|  | Add variety to normal meals | 3 (2.4) |
|  | ^__ b^ | 5 (4.0) |
| **Where the OFD order was delivered, n (%)** | | |
|  | Home | 98 (79.0) |
|  | Workplace | 7 (5.6) |
|  | University or Technical and Further Education | 10 (8.1) |
|  | High school | 0 (0) |
|  | Other | 4 (3.2) |
|  | Recreational center | 1 (0.8) |
|  | Friend/partner’s house | 2 (1.6) |
|  | Library | 1 (0.8) |
|  | ^__ b^ | 5 (4.0) |
| **Distance from food outlet in walking minutes, n (%)** | | |
|  | <15 | 17 (13.7) |
|  | 15-30 | 68 (54.8) |
|  | 30-60 | 27 (21.8) |
|  | >60 | 6 (4.8) |
|  | ^__ b^ | 6 (4.8) |
| **Promotional offer used, n (%)** | | |
|  | Yes | 37 (29.8) |
|  | No | 82 (66.1) |
|  | ^__ b^ | 5 (4.0) |
| **Details on promotions used^c^, n (%)** | | |
|  | Free delivery | 4 (10.8) |
|  | Discount | 30 (91.9) |
|  | 10% off | 3 (8.1) |
|  | 50% off | 3 (8.1) |
|  | $10AUD ($6.56 USD) off | 3 (8.1) |
|  | $15AUD ($9.84 USD) off | 3 (8.1) |
|  | 20% off | 2 (5.4) |
|  | $2AUD ($1.31 USD) off | 2 (5.4) |
|  | $5AUD ($3.28 USD) off | 2 (5.4) |
|  | Via email | 2 (5.4) |
|  | Pizza bundle | 2 (5.4) |
|  | Other (varied)^a^ | 8 (21.6) |
|  | ^__ b^ | 3 (8.1) |
| **Hunger level before ordering, n (%)** | | |
|  | Full | 7 (5.6) |
|  | Quite full | 11 (8.8) |
|  | Not hungry or full | 38 (30.6) |
|  | Quite hungry | 45 (36.2) |
|  | Really hungry | 18 (14.5) |
|  | ^__ b^ | 5 (4.0) |
| **Cravings satisfaction, n (%)** | | |
|  | Yes | 90 (72.5) |
|  | No | 29 (23.4) |
|  | ^__ b^ | 5 (4.0) |
| **Stress levels before ordering, n (%)** | | |
|  | Very stressed | 8 (6.5) |
|  | Stressed | 25 (20.2) |
|  | Not stressed nor relaxed | 57 (46.0) |
|  | Quite relaxed | 17 (13.7) |
|  | Very relaxed | 5 (4.0) |
|  | ^__ b^ | 12 (9.7) |

^a^Please refer to the manuscript for more details.

^b^—Not Available owing to missing values (empty field).

^c^Responses from those that answered ‘Yes’ to using a promotional offer.
